# m^6^A RNA Methylation Regulates the Self-Renewal and Tumorigenesis of Glioblastoma Stem Cells

**DOI:** 10.1016/j.celrep.2017.02.059

**Published:** 2017-03-14

**Authors:** Qi Cui, Hailing Shi, Peng Ye, Li Li, Qiuhao Qu, Guoqiang Sun, Guihua Sun, Zhike Lu, Yue Huang, Cai-Guang Yang, Arthur D. Riggs, Chuan He, Yanhong Shi

**Affiliations:** 1 Division of Stem Cell Biology Research, Department of Developmental and Stem Cell Biology, Beckman Research Institute of City of Hope, Duarte, CA 91010, USA; 2 Irell and Manella Graduate School of Biological Sciences, Beckman Research Institute of City of Hope, Duarte, CA 91010, USA; 3 Department of Chemistry and Department of Biochemistry and Molecular Biology, and Institute for Biophysical Dynamics, Howard Hughes Medical Institute, The University of Chicago, 929 East 57th Street, Chicago, IL 60637, USA; 4 Diabetes and Metabolism Research Institute at City of Hope, Duarte, CA 91010, USA; 5 State Key Laboratory of Drug Research, Shanghai Institute of Materia Medica, Chinese Academy of Sciences, Shanghai 201203, China

## Abstract

RNA modifications play critical roles in important biological processes. However, the functions of *N*^6^-methyladenosine (m^6^A) mRNA modification in cancer biology and cancer stem cells remain largely unknown. Here, we show that m^6^A mRNA modification is critical for glioblastoma stem cell (GSC) self-renewal and tumorigenesis. Knockdown of METTL3 or METTL14, key components of the RNA methyltransferase complex, dramatically promotes human GSC growth, self-renewal, and tumorigenesis. In contrast, overexpression of METTL3 or inhibition of the RNA demethylase FTO suppresses GSC growth and self-renewal. Moreover, inhibition of FTO suppresses tumor progression and prolongs lifespan of GSC-grafted mice substantially. m^6^A sequencing reveals that knockdown of METTL3 or METTL14 induced changes in mRNA m^6^A enrichment and altered mRNA expression of genes (e.g., *ADAM19*) with critical biological functions in GSCs. In summary, this study identifies the m^6^A mRNA methylation machinery as promising therapeutic targets for glioblastoma.

## Graphical abstract

Cui et al. show that m^6^A RNA methylation regulates the self-renewal and tumorigenesis of glioblastoma stem cells (GSCs) by regulating mRNA m^6^A enrichment and expression. An FTO inhibitor suppresses glioblastoma progression and prolongs lifespan of GSC-grafted animals, suggesting that targeting the m^6^A mRNA methylation machinery is a promising therapeutic tool for glioblastoma.

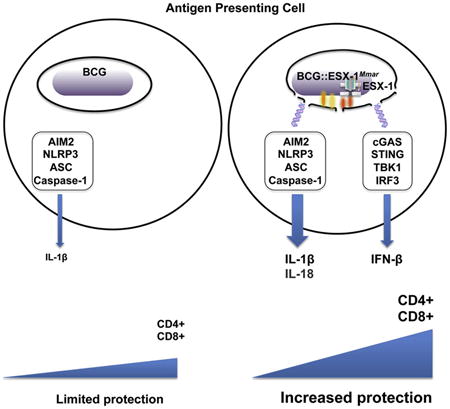

## Introduction

More than 100 RNA modifications have been reported, including modifications within mRNAs (Machnicka et al., 2013), among which *N*^6^-methyladenosine (m^6^A) modification is the most prevalent internal modification in eukaryotic mRNAs (Wei et al., 1975). Although discovered in the 1970s (Desrosiers et al., 1974; Wei and Moss, 1974; Dubin and Taylor, 1975; Perry et al., 1975), the physiological significance of m^6^A modification in mRNA has only been appreciated in recent years because of breakthrough findings of two mammalian RNA demethylases, the fat mass- and obesity-associated protein (FTO) and alkylation repair homolog protein 5 (ALKBH5), which demonstrated that m^6^A methylation is a dynamic and reversible modification (Jia et al., 2011; Zheng et al., 2013). Transcriptome-wide m^6^A profiling further showed that m^6^A modification is presented in thousands of RNA transcripts with unique distribution patterns (Dominissini et al., 2012; Meyer et al., 2012).

The formation of m^6^A modification is catalyzed by a methyltransferase complex that contains methyltransferase-like 3 (METTL3), methyltransferase-like 14 (METTL14), and Wilm's-tumor-1-associated protein (WTAP) in mammalian cells (Bokar et al., 1994; Liu et al., 2014; Ping et al., 2014; Wang et al., 2014). Knockdown (KD) of either METTL3 or METTL14 induces a substantial decrease in m^6^A levels in mRNA (Liu et al., 2014; Wang et al., 2014). While m^6^A methyltransferases and demethylases act as its writers and erasers, respectively, m^6^A readers selectively bind to m^6^A-containing RNA to mediate downstream effects (Yue et al., 2015).

The roles of RNA modifications in biological processes have just begun to be appreciated. RNA modifications have been implicated in embryonic stem cell maintenance and differentiation (Batista et al., 2014; Wang et al., 2014; Geula et al., 2015), circadian rhythm modification (Fustin et al., 2013), heat shock response (Zhou et al., 2015), meiotic progression (Schwartz et al., 2013), and neuronal function (Lemkine et al., 2005). However, the function of the majority of RNA modifications found in mRNAs remains unknown. Specifically, the functional roles of m^6^A methylation in cancer initiation and progression remain to be determined. The identification of the writers, readers, and erasers of m^6^A modification and the development of the m^6^A-sequencing (m^6^A-seq) technology set the foundation for the field to define the roles of m^6^A mRNA modification in cancer biology.

Glioblastoma is the most deadly primary brain tumor. Even with the combined surgical resection, radiation therapy, and chemotherapy, median survival of patients is less than 15 months after diagnosis (Stupp et al., 2009; Johnson and O'Neill, 2012). Lack of success in treating glioblastoma likely arises from tumor heterogeneity and treatment resistance of glioblastoma stem cells (GSCs), a population of cancer stem cells with an extraordinary capacity to promote tumor growth and invasion that display increased resistance to radiotherapy and chemotherapy (Singh et al., 2004; Bao et al., 2006; Godlewski et al., 2010). The presence of these cancer stem cells renders glioblastoma treatment resistant and recurring (Sundar et al., 2014). Therefore, new glioblastoma therapies that target these treatment-resistant cancer stem cells are urgently needed (Godlewski et al., 2010; Allegra et al., 2014).

In this study, we demonstrate that KD of METTL3 or METTL14 expression dramatically increased GSC growth and self-renewal. In contrast, overexpression of METTL3 or treatment with MA2, a chemical inhibitor of the RNA demethylase FTO, inhibited GSC growth and self-renewal considerably. By transplanting METTL3 small hairpin RNA (shRNA) or METTL14 shRNA-transduced GSCs into immunodeficient non-obese diabetic (NOD) severe combined immunodeficiency (SCID) gamma (NSG) mice, we show that KD of METTL3 or METTL14 expression led to substantial increase of GSC-initiated tumor progression in transplanted mouse brains. Furthermore, treatment with MA2, a chemical inhibitor of FTO, dramatically suppressed GSC-induced tumorigenesis and prolonged lifespan in GSC-grafted animals.

## Results

### m^6^A Levels in GSCs Are Elevated upon Induced Differentiation

**Figure 1:**
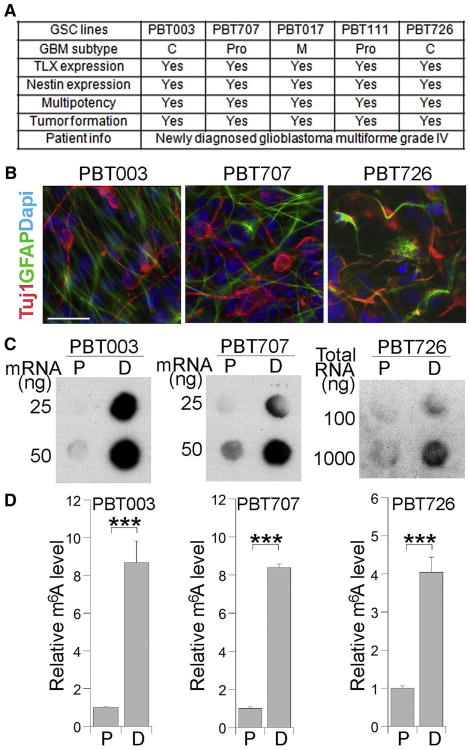
Differentiation of GSCs Induces Elevated Levels of m^6^A RNA Modification (A) A list of GSC lines used in this study. The characterization of these GSCs, including glioblastoma (GBM) subtype, marker (TLX and nestin) expression, multipotency, and tumor-formation capacity, is summarized in the table. (B) Differentiation of GSCs into Tuj1-positive neurons (red) and GFAP-positive astrocytes (green) by treating cells with FBS together with retinoic acid. Scale bar, 25 μm. (C) RNA dot blot analysis of m^6^A levels in proliferating (P) GSCs and differentiated (D) cells. (D) Quantification of m^6^A level measured by RNA dot blot shown in (C). n = 4. ***p < 0.001 by Student's t test. Error bars represent SD of the mean.

Primary GSCs were isolated from tumor tissues of newly diagnosed World Health Organization (WHO) grade IV glioblastoma patients and cultured as 3D tumorspheres in a culture condition optimized for GSC enrichment (Brown et al., 2009). We included five GSC lines that represent different glioblastoma subtypes in this study. Among these GSC lines, PBT003 and PBT726 are classical (C), PBT707 and PBT111 are proneural (Pro), and PBT017 is mesenchymal (M) (Cui et al., 2016). These GSCs expressed neural stem cell markers and exhibited multipotency, having the ability to give rise to both neurons and astrocytes. Moreover, they could form brain tumors with typical glioblastoma features in transplanted mouse brains as we described previously (Cui et al., 2016). A summary of these cell lines is included in the table in Figure 1A. To determine the relationship between cellular differentiation of GSCs and m^6^A modification, we induced three lines of GSCs (PBT003, PBT707, and PBT726) into differentiation using fetal bovine serum (FBS) together with retinoic acid as previously described (Lang et al., 2012). The differentiation of GSCs into neurons and astrocytes was confirmed by immunostaining using antibodies specific for the neuronal marker βIII tubulin (Tuj1) and the astrocyte marker GFAP (Figure 1B). The level of m^6^A in differentiated (D) cells was measured by m^6^A mRNA dot blot and compared to that in proliferating (P) GSCs. Dramatically elevated m^6^A level was detected in GSCs that were induced into differentiation, compared to GSCs that were proliferating (Figures 1C and 1D). These results indicate that m^6^A levels are dynamically regulated when GSCs are induced into differentiation.

### KD of METTL3 or METTL14 Enhances GSC Growth and Self-Renewal

**Figure 2:**
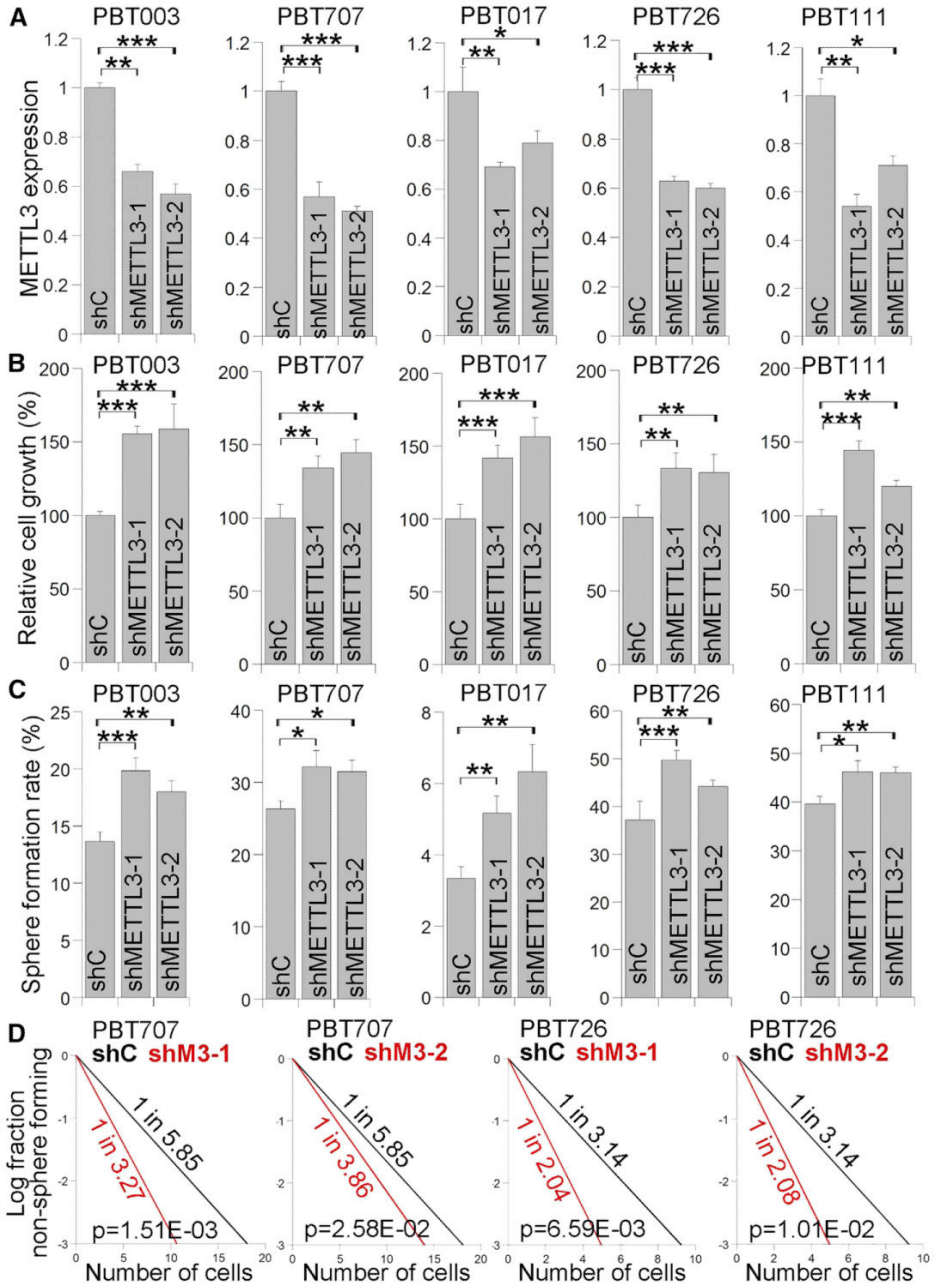
Knocking Down METTL3 Expression Promotes the Growth and Self-Renewal of GSCs (A) RT-PCR analysis of METTL3 expression in GSCs transduced with lentivirus expressing control shRNA (shC) or METTL3 shRNAs (shMETTL3-1, shMETTL3-2). n = 3. See also Figure S1. (B–D) Cell growth (B), sphere-formation (C), and limiting dilution assay (LDA) (D) of GSCs transduced with lentivirus expressing control shRNA or METTL3 shRNAs. A sphere-formation assay and LDA were used to evaluate the self-renewal capacity of GSCs. n = 4 for (B), n = 6 for (C), and n = 20 for (D). *p < 0.05, **p < 0.01, and ***p < 0.001 by Student's t test. Error bars represent SD of the mean.

To determine if m^6^A modification plays a role in GSC self-renewal and tumorigenesis, we knocked down METTL3, the catalytic subunit of m^6^A methyltransferase complex (Bokar et al., 1997; Batista et al., 2014; Liu et al., 2014; Wang et al., 2014; Geula et al., 2015), using two distinct shRNAs in the five lines of GSCs (PBT003, PBT707, PBT017, PBT726, and PBT111). KD of METTL3 expression by both shRNAs was confirmed by RT-PCR (Figure 2A) and western blot (Figure S1). Reduced mRNA m^6^A levels in METTL3 KD cells were confirmed by mRNA dot blot (Figure S1). KD of METTL3 increased cell growth substantially in all GSC lines tested (Figure 2B). Moreover, KD of METTL3 enhanced the self-renewal of these GSC lines considerably, as revealed by the significantly increased sphere-formation rate and stem cell frequency in METTL3 KD GSCs (Figures 2C and 2D). Accordingly, the expression of CD44, a GSC marker (Anido et al., 2010; Pietras et al., 2014), was upregulated in METTL3 KD GSCs (Figure S1).

**Figure 3:**
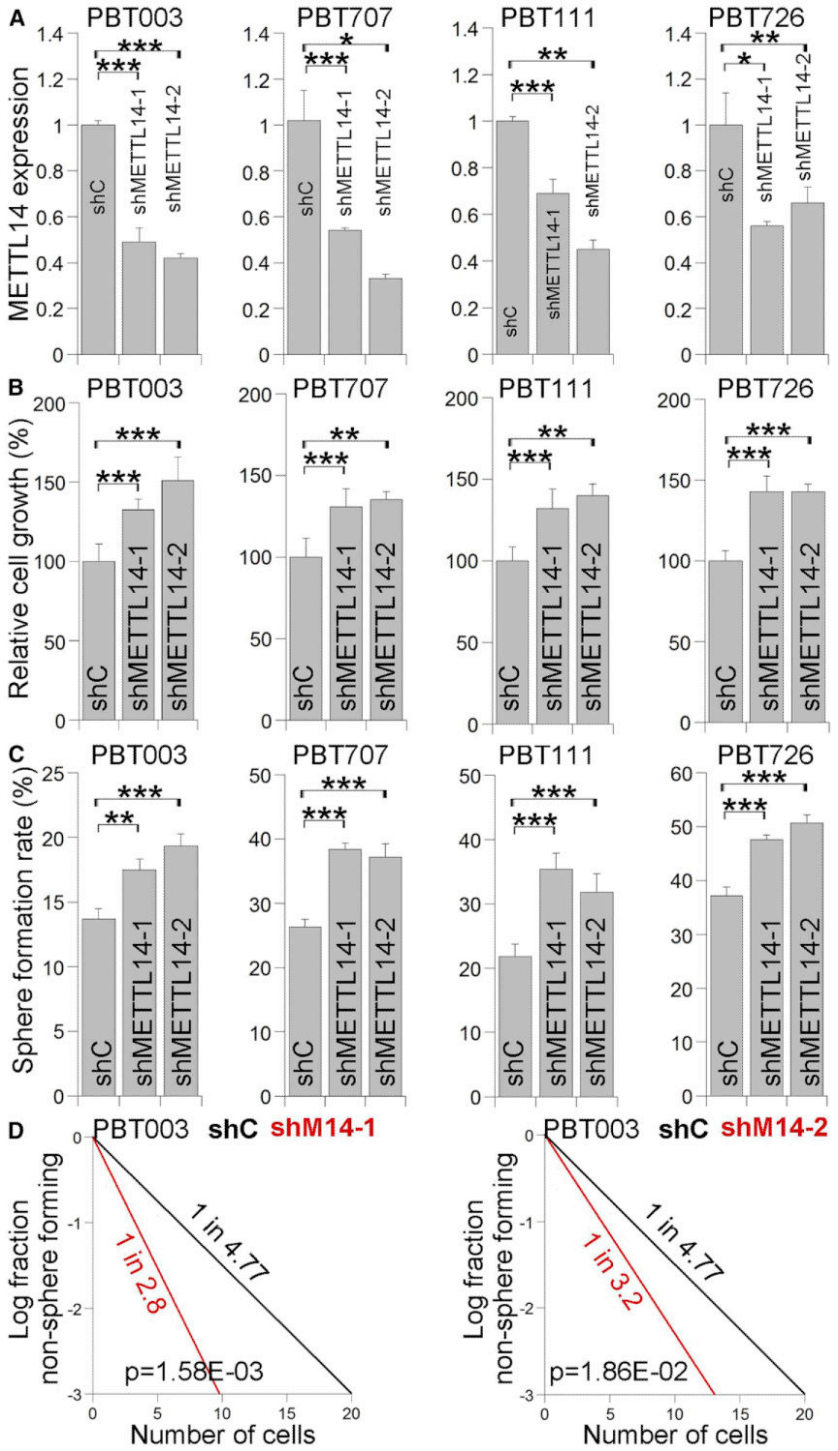
Knocking Down METTL14 Expression Enhances the Growth and Self-Renewal of GSCs (A) RT-PCR analysis of METTL14 expression in GSCs transduced with lentivirus expressing control shRNA (shC) or METTL14 shRNAs (shMETTL14-1, shMETTL14-2). n = 3. See also Figure S1. (B–D) Cell growth (B), sphere-formation (C), and LDA (D) analyses of GSCs transduced with lentivirus expressing control shRNA or METTL14 shRNAs. n = 4 for (B), n = 6 for (C), and n = 20 for (D). *p < 0.05, **p < 0.01, ***p < 0.001 by Student's t test. Error bars represent SD of the mean.

METTL14 is another component of the methyltransferase complex that is critical for m^6^A methylation (Liu et al., 2014; Ping et al., 2014; Schwartz et al., 2014; Wang et al., 2014). Like METTL3 KD, METTL14 KD has also been shown to reduce mRNA m^6^A levels (Liu et al., 2014; Schwartz et al., 2014; Wang et al., 2014). To further determine the role of mRNA m^6^A methylation in GSCs, we modified mRNA m^6^A levels by knocking down METTL14 using two distinct METTL14 shRNAs in GSCs, including PBT003, PBT707, PBT111, and PBT726. KD of METTL14 was confirmed by RT-PCR (Figure 3A) and western blot (Figure S1), and reduced mRNA m^6^A levels in METTL14 KD cells were revealed by mRNA dot blot (Figure S1). Similar to KD of METTL3, KD of METTL14 elevated CD44 expression (Figure S1) and enhanced the growth and self-renewal of GSCs substantially (Figures 3B–3D). These results together indicate that reduced level of mRNA m^6^A modification promotes GSC growth and self-renewal.

### Overexpressing METTL3 Inhibits GSC Growth and Self-Renewal

**Figure 4:**
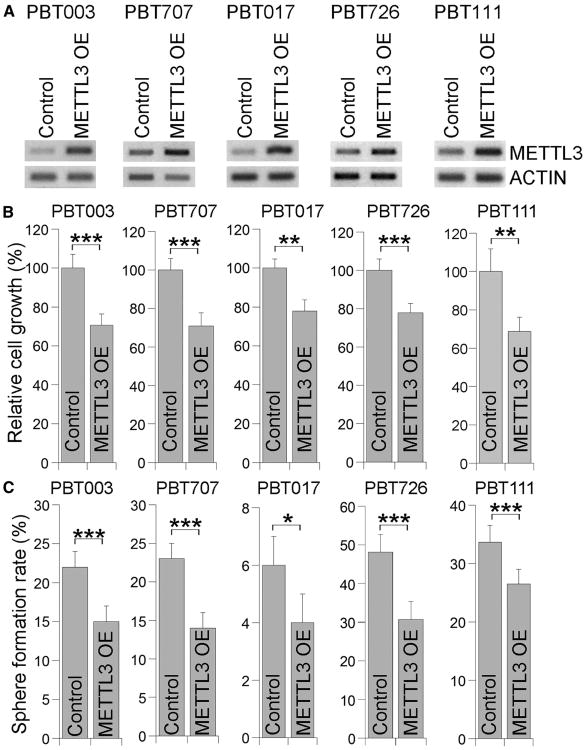
Overexpressing METTL3 Inhibits the Growth and Self-Renewal of GSCs (A) RT-PCR analysis showing overexpression of METTL3 in GSCs. (B and C) Cell growth (B) and sphere-formation (C) analyses of GSCs transduced with METTL3-expressing virus or control virus. n = 4 for (B) and n = 6 for (C). *p < 0.05, **p < 0.01, and ***p < 0.001 by Student's t test. Error bars represent SD of the mean. See also Figure S2.

In addition to decreasing m^6^A level by KD of METTL3 or METTL14, we next investigated how increased m^6^A levels affect the growth and self-renewal of GSCs by overexpressing METTL3. Multiple GSC lines, including PBT003, PBT707, PBT017, PBT726, and PBT111, were transduced with lentivirus expressing control vector or METTL3. mRNAs were isolated from control or METTL3-overex-pressing cells, and RT-PCR analysis confirmed the overexpression of METTL3 in GSCs transduced with METTL3-expressing lentivirus (Figure 4A). Elevated m^6^A levels were detected in METTL3-overexpressing GSCs as expected (Figure S1). Overexpression of METTL3 reduced the growth and self-renewal in all GSC lines tested (Figures 4B and 4C). Reduced expression of CD44 was also observed in METTL3-overexpressing GSCs (Figure S1). In contrast, overexpression of a catalytically inactive mutant of METTL3 (Lin et al., 2016) had minimal effect on GSC growth and self-renewal (Figures S2A–S2D). Moreover, expression of the catalytically inactive METTL3 failed to reverse the elevated sphere-formation pheno-type induced by METTL3 KD, whereas expression of wild-type METTL3 was able to rescue the phenotype (Figure S2E). These results together indicate that METTL3 regulates GSC growth and self-renewal through its methyltransferase catalytic activity.

### KD of METTL3 or METTL14 Promotes Tumor Progression

**Figure 5:**
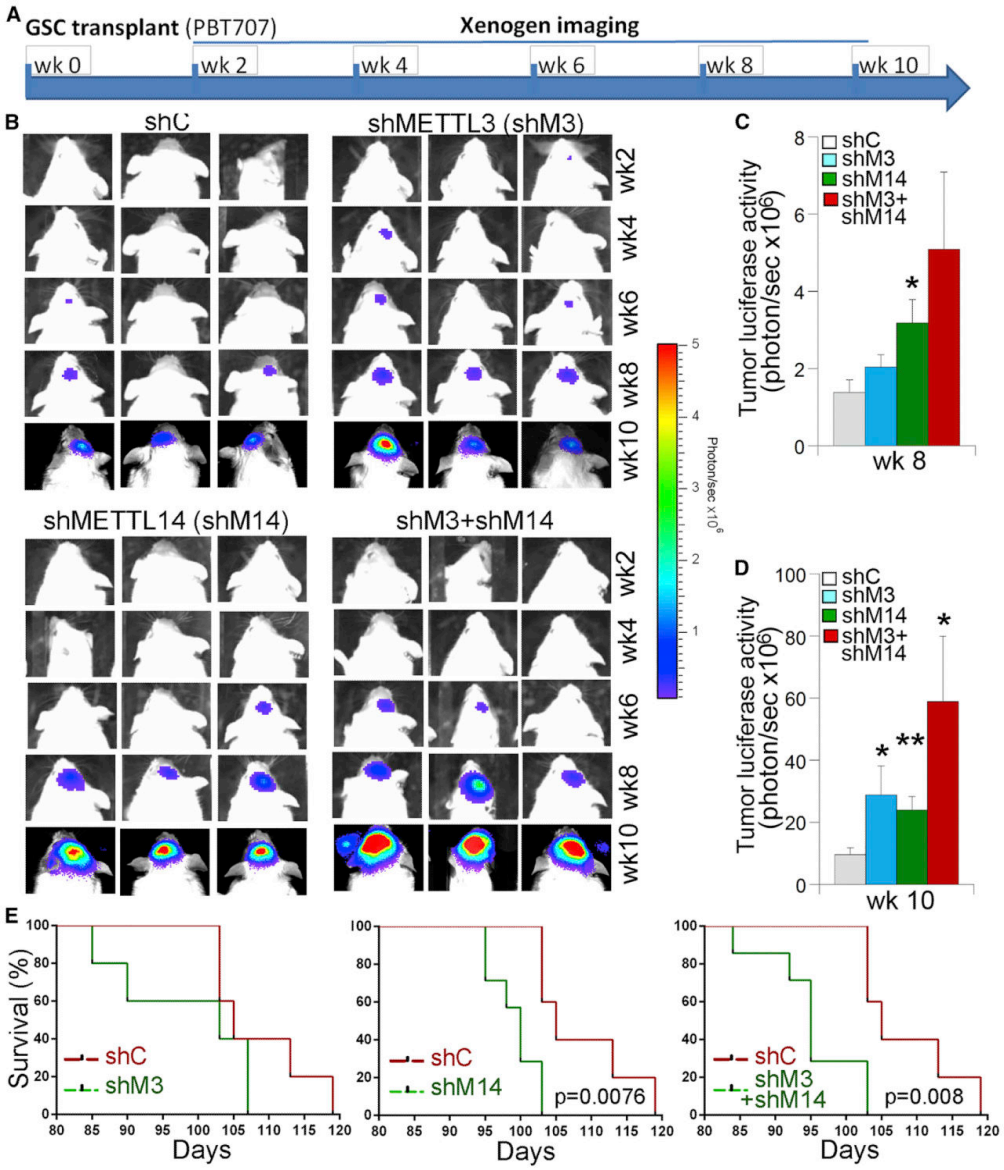
Knocking Down METTL3 and/or METTL14 Expression Promotes the Tumorigenicity of GSCs (A) Schematic of the experimental design, including GSC transplantation and xenogen imaging of xenografted tumors. (B) Xenogen images of brain tumors in NSG mice transplanted with PBT707 cells that were transduced with control shRNA (shC), METTL3 shRNA (shMETTL3), or METTL14 shRNA (shMETTL14). The scale bar for bioluminescence intensity is shown on the right. (C and D) Quantification of the bioluminescence intensity of tumors at 8 weeks (C) and 10 weeks (D) after tumor transplantation. *p < 0.05 and **p < 0.01 by Student's t test. Error bars represent SD of the mean. (E) The survival curves of NSG mice transplanted with PBT707 cells transduced with control shRNA (shC), METTL3 shRNA (shMETTL3), or METTL14 shRNA (shMETTL14). The x axis represents days after GSC transplantation. n = 7, log-rank test. See also Figures S3–S5.

The dramatic effect of METTL3 or METTL14 KD on GSC growth and self-renewal in vitro prompted us to test whether KD of METTL3 or METTL14 affects the ability of GSCs to form tumors in vivo. Luciferase-expressing PBT707 cells were transduced with lentivirus expressing a control shRNA, a METTL3 shRNA, or a METTL14 shRNA. The transduced cells were orthotopically transplanted into the frontal lobe of NSG mouse brains (Figure 5A). Tumor formation was monitored by bioluminescence xenogen imaging (Figure 5B). Compared to mice receiving control-shRNA-transduced GSCs (control GSCs), mice grafted with METTL3 KD GSCs exhibited much bigger tumors, as revealed by a substantial increase in tumor bioluminescence intensity (Figures 5B–5D). Likewise, mice grafted with METTL14 KD GSCs also exhibited dramatically bigger tumors than mice grafted with control GSCs (Figures 5B–5D). In addition to single KD of METTL3 or METTL14, we transduced PBT707 cells with a mixture of lentivirus containing both METTL3 shRNA and METTL14 shRNA at a dose, when combined, that was similar to that used in single-KD experiments. Mice grafted with the GSCs with KD of both METTL3 and METTL14 resulted in an increase in tumor progression that was even more dramatic than that seen in mice grafted with GSCs with METTL3 or METTL14 KD alone (Figures 5B–5D).

Consistent with the aggressive tumor progression, mice grafted with PBT707 cells with KD of METTL3 or METTL14 alone or KD of both METTL3 and METTL14 had considerably worse survival outcomes than mice grafted with control GSCs (Figure 5E). These results together indicate that inhibition of m^6^A RNA methylation by knocking down METTL3 and/or METTL14 promotes tumor progression and shortens the lifespan of GSC-grafted animals.

Similar to what was observed for the PBT707 cell line, when luciferase-expressing PBT003 cells were transduced with METTL3 shRNA and then transplanted into the brains of NSG mice (Figure S3A), a dramatic increase in tumor progression, as revealed by elevated tumor luciferase activity, was detected when compared to that in mice transplanted with PBT003 cells transduced with a control shRNA (Figures S3B and S3C). Moreover, mice transplanted with METTL3 KD PBT003 cells exhibited an overall shorter lifespan than mice transplanted with control PBT003 cells (Figure S3D).

In addition to KD of METTL3, we also transduced PBT003 cells with lentivirus expressing a METTL14 shRNA or the combination of METTL3 and METTL14 shRNAs. The transduced cells were transplanted into the brains of NSG mice, and tumor progression was monitored by xenogen imaging (Figure S4A). Compared to mice grafted with control cells, mice transplanted with PBT003 cells having METTL14 KD or METTL3 and METTL14 double KD developed much bigger tumors, as revealed by a dramatic increase in tumor luciferase activity at week 4, 5, or 6 after GSC transplantation (Figures S4B and S4C). Moreover, mice transplanted with PBT003 cells with both METTL3 and METTL14 KD exhibited significantly worse survival outcome and much shorter overall lifespan than mice transplanted with control cells (Figure S4D).

Likewise, when we transplanted PBT726 cells with KD of METTL3 or METTL14 into the brains of NSG mice, we observed a substantial increase in tumor growth compared to that in mice transplanted with control cells (Figure S5). These results further support the idea that inhibiting m^6^A methylation in GSCs by knocking down METTL3 or METTL14 promotes tumor progression.

### The FTO Inhibitor MA2 Inhibits Tumor Progression

To evaluate the efficacy of modifying m^6^A levels in GSC tumorigenesis in a more clinically relevant system, we transplanted luciferase-reporter-bearing PBT003 cells into brains of NSG mice to establish tumors and treated the mice with an FTO inhibitor that has been shown to modulate mRNA m^6^A levels (Huang et al., 2015). FTO was identified as the first RNA demethylase that oxidatively demethylates m^6^A in mRNAs (Jia et al., 2011). MA2, the ethyl ester form of meclofenamic acid (MA), a US Food and Drug Administration (FDA)-approved nonsteroidal anti-inflammatory drug, was recently identified as a selective inhibitor of FTO that increases m^6^A levels in mRNA of human cells (Huang et al., 2015). Indeed, a substantial increase in mRNA m^6^A levels was detected in GSCs treated with 50 μM MA2, as shown by m^6^A mRNA dot blot analysis (Figure S1).

**Figure 6:**
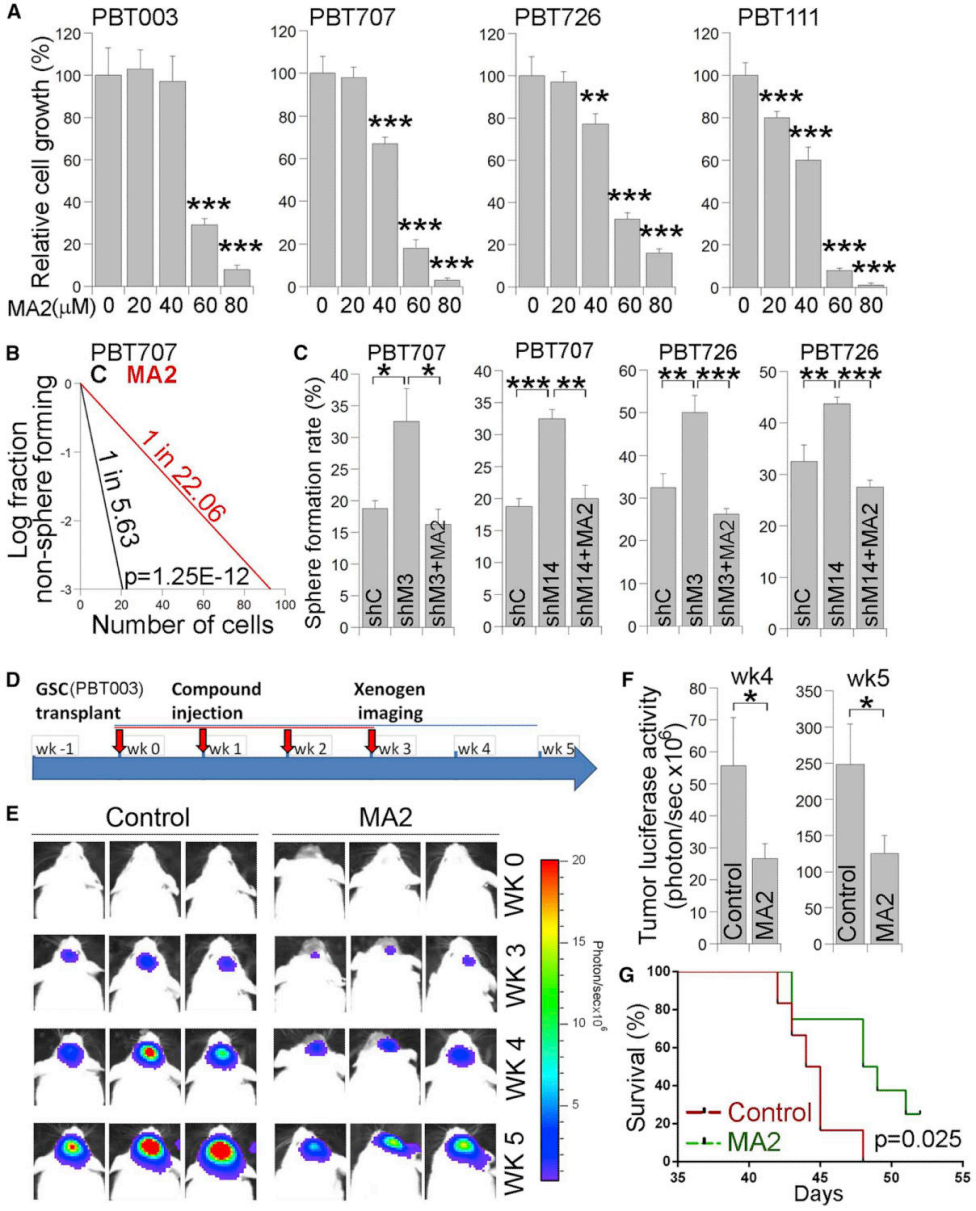
Treatment with the FTO Inhibitor MA2 Reduces GSC-Initiated Tumor Growth (A) Cell growth analyses of GSCs treated with the FTO inhibitor MA2. n = 4. (B) LDA analysis of GSCs treated with MA2 or vehicle control. n = 20. (C) Sphere-formation analysis of GSCs treated with control shRNA (shC) or METTL3 or METTL14 shRNA (shM3 or shM14) expressing virus alone or together with MA2. *p < 0.05, **p < 0.01, and ***p < 0.001 by Student's t test. Error bars represent SD of the mean. See also Figure S6. (D) Schematic of the experimental design, including GSC transplantation, MA2 treatment, and xenogen imaging of tumors derived from grafted GSCs. The transplanted mice were treated with the FTO inhibitor MA2 or vehicle control. (E) Xenogen images of brain tumors in GSC-grafted NSG mice treated with vehicle control (C) or MA2. The scale bar for bioluminescence intensity is shown on the right. (F) Quantification of the bioluminescence intensity of tumors. n = 10. *p < 0.05 by Student's t test. Error bars represent SD of the mean. (G) The survival curves of GSC-grafted NSG mice treated with MA2 or vehicle control. The x axis represents days after the first MA2 treatment. n = 8, log-rank test.

Next, we tested the effect of MA2 treatment on GSC growth and self-renewal in vitro. A dramatic growth inhibitory effect of MA2 was detected in PBT003 cells at 60 μM and 80 μM doses (Figure 6A). MA2 also exerted a substantial inhibitory effect on the growth of other GSC lines, including PBT707, PBT726 and PBT111, at the same doses (Figure 6A). In addition, MA2 suppressed the growth of PBT707 and PBT726 at a lower dose of 40 μM and inhibited the growth of PBT111 at lower doses of 20 μM and 40 μM(Figure 6A). In contrast, no substantial effect was detected on the growth of the normal neural stem cell (NSC) line NSC006, brain astrocytes, or HeLa cells when MA2 was used at doses up to 60 μM(Figure S6A). A mild effect was observed in NSCs, astrocytes, or HeLa cells treated with 80 μM MA2 (Figure S6A). In addition to suppressing GSC growth, MA2 treatment dramatically inhibited the self-renewal of GSCs as revealed by reduced stem cell frequency in MA2-treated GSCs compared to control cells (Figures 6B and S6B). Moreover, MA2 treatment reversed the effect of elevated sphere-formation rates induced by METTL3 or METTL14 KD in GSCs (Figure 6C). The results of MA2 treatment corroborated our observation in GSCs overexpressing METTL3, further strengthening our hypothesis that an increase in m^6^A levels inhibits GSC growth and self-renewal.

To test the effect of the FTO inhibitor on GSC-initiated tumorigenesis, we treated PBT003-grafted mice with the selective FTO inhibitor MA2 intratumorally once a week for 4weeks (Figure 6D). Tumor formation was monitored by bioluminescence xenogen imaging. Compared to mice receiving vehicle control, mice treated with MA2 had much smaller tumors (Figure 6E). Bioluminescence measurement showed a significant decrease of tumor luciferase activity in mice treated with MA2 at 4 or 5 weeks after compound treatment (Figure 6F). Consistent with reduced tumor growth, mice treated with MA2 had substantially prolonged survival compared to mice treated with vehicle control (Figure 6G). This result indicates that small-molecule compounds that increase m^6^A RNA methylation have therapeutic potential to inhibit GSC tumorigenesis.

### KD of METTL3 or METTL14 Alters Gene Expression in GSCs

**Figure 7:**
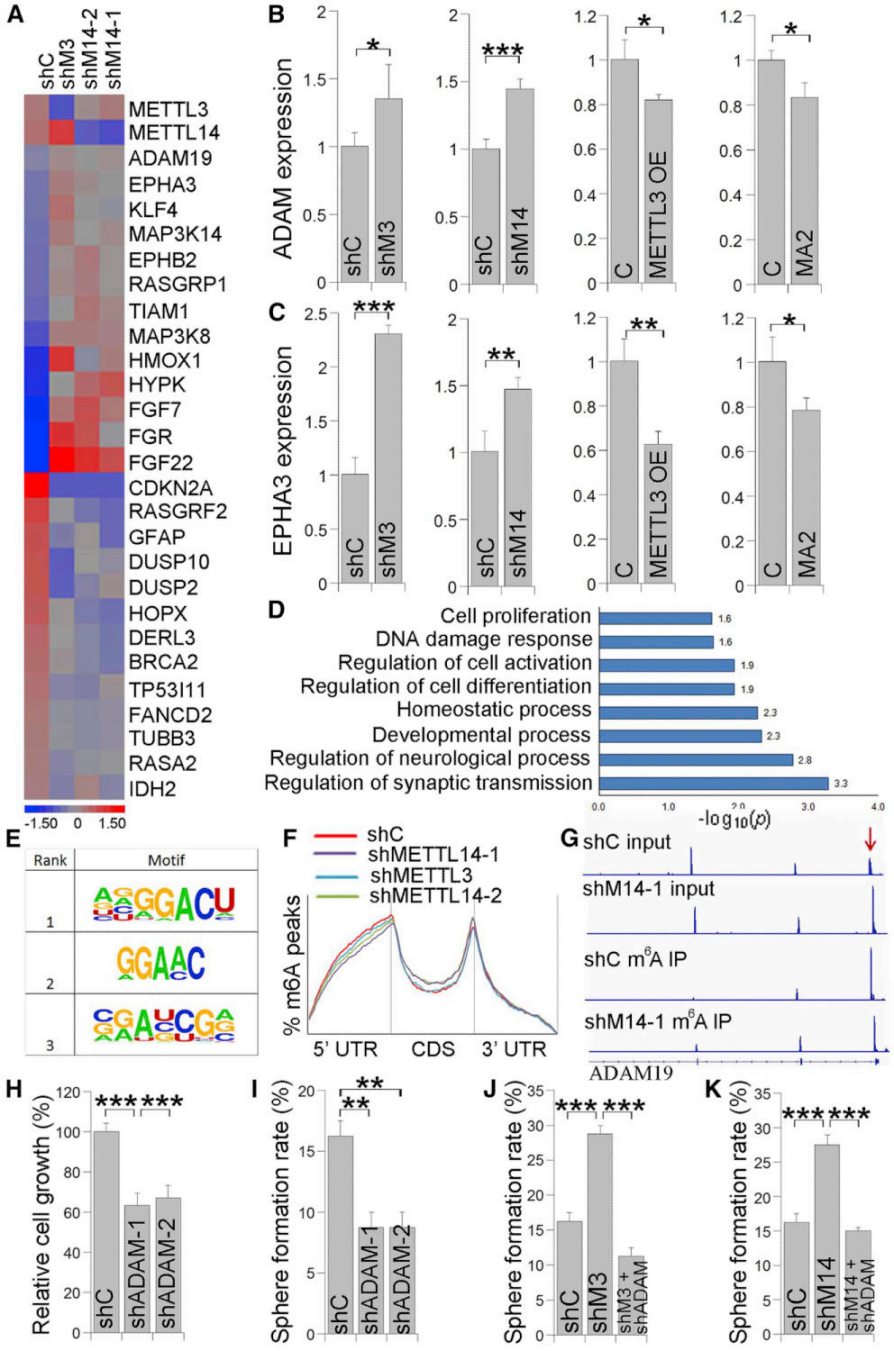
METTL3 or METTL14 KD Induces mRNA Expression and m^6^A Methylation Level Change in GSCs A) Heatmap showing mRNA expression changes in PBT003 cells with METTL3 or METTL14 KD. shC, control shRNA; shM3, shRNA for METTL3; shM14-1 and shM14-2, shRNAs for METTL14. (B and C) RT-PCR of ADAM19 (ADAM) (B) and EPHA3 (C) expression in PBT003 cells with METTL3 or METTL14 KD, METTL3 overexpression (OE), or MA2 treatment. n = 3. See also Figure S7. (D) GO analysis of genes with expression change upon METTL3 or METTL14 KD in PBT003 cells. (E and F) The m^6^A motif (E) and peak distribution (F) in GSCs. (G) Change of the m^6^A methylation level in ADAM19 mRNA in PBT003 cells with METTL14 KD. The mRNA input in shC and shM14-1 cells is included in the top rows. mRNA pulled down by immunoprecipitation with an m^6^A antibody (m^6^A IP) is included in the bottom rows. (H and I) Cell growth (H) and sphere-formation (I) analyses of PBT003 cells transduced with lentivirus expressing shC or ADAM19 shRNAs (shADAM-1, shADAM-2). n = 4. (J and K) Sphere-formation assay of PBT003 cells transduced with lentivirus expressing METTL3 shRNA (J) or METTL14 shRNA (K) alone or together with ADAM19 shRNA (shADAM). n = 4. *p < 0.05, **p < 0.01, and ***p < 0.001 by Student's t test. Error bars represent SD of the mean. See also Figure S7.

To investigate the mechanism underlying how m^6^A modification regulates GSC tumorigenesis, RNA sequencing (RNA-seq) was performed to detect gene expression changes in PBT003 cells with KD of METTL3 or METTL14. The expression of more than 2,600 transcripts was changed in PBT003 cells with METTL3 or METTL14 KD compared to control cells. Among the genes with altered expression, a number of oncogenes, such as *ADAM19*, *EPHA3*, and *KLF4*, were upregulated, and many tumor suppressors, such as *CDKN2A*, *BRCA2*, and *TP53I11*, were downregulated in GSCs with KD of METTL3 or METTL14 (Figure 7A). The expression of differentiated neural cell markers, such as the astrocyte marker GFAP and the neuronal marker TUBB3 (Tuj1), was also decreased in METTL3 or METTL14 KD GSCs (Figure 7A). These gene expression changes were consistent with our hypothesis that KD of METTL3 or METTL14 promotes GSC self-renewal and tumorigenesis.

The upregulated expression of oncogenes, such as *ADAM19*, *EPHA3*, and *KLF4*, in METTL3 or METTL14 KD GSCs was confirmed by RT-PCR. In contrast, overexpression of METTL3 or treatment with the FTO inhibitor MA2 led to decreased expression of these genes (Figures 7B, 7C, and S7A). The altered expression of these oncogenes by perturbation of m^6^A modification suggests that the expressions of these genes are regulated by m^6^A RNA methylation. Gene ontology (GO) analysis revealed that METTL3 or METTL14 KD regulated the expression of genes involved in important biological processes, including cell proliferation, differentiation, and DNA damage response (Figure 7D). Taken together, these data indicate that mRNA m^6^A modification can regulate GSC tumorigenesis by controlling the expression of cancer-associated genes and processes.

### m^6^A-Modified mRNAs Are Involved in Critical Cellular Processes

To investigate the m^6^A modifications in the GSC transcriptome, m^6^A-seq analysis was performed as described previously (Dominissini et al., 2013). The m^6^A consensus motif GGAC was identified in PBT003 cells (Figure 7E). Peak distribution analysis revealed strong enrichment of m^6^A peaks near the stop codon (Figure 7F) as previously described (Dominissini et al., 2012; Meyer et al., 2012). Of interest, strong enrichment of m^6^A peaks was also detected near the start codon in GSCs (Figure 7F). GO analysis of genes with m^6^A peaks in their mRNAs revealed that m^6^A-methylated mRNAs are involved in critical cellular processes, such as cell growth, cell differentiation, DNA damage response, and cellular stress response (Figure S7B).

Several genes in which mRNA has been modified by m^6^A methylation play critical roles in cell growth and tumorigenesis. For example, the mRNA of *ADAM19*, a metalloproteinase disintegrin gene that exhibits elevated expression in glioblastoma cells and promotes glioblastoma cell growth and invasiveness (Wildeboer et al., 2006; Mochizuki and Okada, 2007), is m^6^A methylated. mRNA expression of *ADAM19* is highly elevated in METTL14 KD GSCs, as revealed by increased mRNA reads in METTL14 KD GSCs (shM14-1 input), compared to control GSCs (shC input) (Figure 7G). In contrast, m^6^A enrichment in the mRNA of this gene is dramatically reduced upon KD of METTL14, as revealed by the reduced mRNA m^6^A peak in METTL14 KD GSCs (shM14-1 m^6^A IP), compared to control GSCs (shC m^6^A IP) (Figure 7G), correlating with upregulated expression of this gene by KD of METTL14. KD of ADAM19 dramatically reduced the growth and self-renewal of GSCs (Figures 7H, 7I, and S7). Moreover, the elevated sphere-formation rate induced by KD of METTL3 or METTL14 in GSCs could be reversed by KD of ADAM19 (Figures 7J, 7K, and S7), suggesting that ADAM19 acts as a target of m^6^A RNA methylation to regulate GSC self-renewal. Taken together, these data demonstrate that mRNA m^6^A methylation is an important RNA epigenetic marker that is involved in regulating the expression of genes with important biological functions in GSCs.

## Discussion

This study demonstrates that controlling mRNA m^6^A level is critical for maintaining GSC growth, self-renewal, and tumor development. KD of METTL3 or METTL14 expression reduced mRNA m^6^A levels, enhanced the growth and self-renewal of GSCs in vitro, and promoted the ability of GSCs to form brain tumors in vivo. In contrast, overexpression of METTL3 or treatment with the FTO inhibitor MA2 increased mRNA m^6^A levels in GSCs and suppressed GSC growth. Moreover, treatment of GSCs with the FTO inhibitor MA2 suppressed GSC-initiated tumorigenesis and prolonged the lifespan of GSC-engrafted mice. Our finding that the FTO inhibitor MA2 suppresses GSC-initiated brain tumor development suggests that m^6^A methylation could be a promising target for anti-glioblastoma therapy.

This study uncovered a critical role for mRNA m^6^A modification in regulating GSC self-renewal and tumorigenesis. Study of mRNA modification is a nascent field as yet, and the significance of this epigenetic mark in controlling cell growth and differentiation is just beginning to be appreciated. Although m^6^A is most abundant in the brain (Meyer et al., 2012), no study on the role of m^6^A modification in either brain development or brain disorders has been reported previously, although recent studies have demonstrated a role for m^6^Ain *Drosophila* neuronal function (Haussmann et al., 2016; Lence et al., 2016). Moreover, the role of m^6^A in cancer is only starting to be revealed (Zhang et al., 2016; Li et al., 2017). This report provides a causative link between mRNA m^6^A methylation and glioblastoma tumorigenesis, which represents an important step toward developing therapeutic strategies to treat glioblastoma by targeting m^6^A modification, its upstream regulators, or its downstream targets in GSCs.

RNA epigenetics has become a fast-moving research field in biology and holds great promise for future therapeutic development for human diseases. The m^6^A modification produced by a methyltransferase complex consisting of METTL3 and METTL14 is one of the most common and abundant mRNA modifications in eukaryotes. The evidence is clear that m^6^A methylation is more than a mere “decoration” of mRNA. The reversible nature of m^6^A methylation strongly suggests a regulatory role for this RNA modification (Sibbritt et al., 2013). Such a role could be important during dynamic cell growth and differentiation processes. Indeed, a role for m^6^A modification in controlling embryonic stem cell pluripotency and differentiation has been reported (Batista et al., 2014; Wang et al., 2014; Chen et al., 2015; Geula et al., 2015). Although components of the m^6^A methylation machinery have been linked to cancer (Linnebacher et al., 2010; Kaklamani et al., 2011; Pierce et al., 2011; Machiela et al., 2012; Long et al., 2013; Lin et al., 2016; Zhang et al., 2016), whether the effect is dependent on m^6^A modification remains to be clarified. A recent study demonstrated that METTL3 enhances translation in cancer cells independently of m^6^A modification (Lin et al., 2016). On the other hand, elevated levels of the S-adenosyl methionine (SAM) donor of the methyl group in the m^6^A methylation process have been shown to suppress cell growth in cancer (Pascale et al., 2002; Pakneshan et al., 2004; Guruswamy et al., 2008; Lu et al., 2009; Zhao et al., 2010). However, whether the growth-inhibitory effect of increased levels of SAM is caused by elevated levels of m^6^A modification remains unknown. A direct causative link between mRNA m^6^A methylation and tumorigenesis remains to be established (Sibbritt et al., 2013). This study revealed the biological significance of m^6^A modification in glioblastoma biology, defining the role of m^6^A modification in GSC self-renewal and tumorigenesis by targeting multiple components of the m^6^A regulatory machinery, including METTL3, METTL14, and FTO.

This study identified key roles of m^6^A modification in glioblastoma, the most aggressive and invariably lethal brain tumor. We focused on GSCs, which are implicated in the initiation and development of glioblastoma. Our results demonstrate that modulation of mRNA m^6^A levels impacts multiple aspects of GSCs, including GSC growth, self-renewal, and tumorigenesis, suggesting that mRNA m^6^A modification may serve as a promising target for GSCs. Furthermore, we performed m^6^A-seq analysis in GSCs with KD of METTL3 or METTL14. This transcriptome-wide analysis revealed genes and pathways that are impacted by mRNA m^6^A modification in GSCs, which could also serve as potential molecular targets to inhibit GSC tumori-genesis in the treatment of glioblastoma.

## Experimental Procedures

### Cell Culture

GSCs derived from patients that were newly diagnosed as grade IV glioblastoma were maintained in sphere cultures as previously described (Cui et al., 2016). Briefly, GSCs were cultured in DMEM-F12 medium (Omega Scientific) supplemented with 1 × B27 (Invitrogen), 2 mM L-glutamine (Media Tech), 27.4 mM HEPES (Fisher Scientific), and growth factors, including 20 ng/mL epidermal growth factor (EGF) (PeproTech), 20 ng/mL fibroblast growth factor (FGF) (PeproTech), and 5 μg/mL heparin (Sigma). All cultures were confirmed for no contamination of mycoplasma using MycoAlert PLUS Mycoplasma Detection Kit (Lonza).

### Plasmid DNA

shRNAs were cloned into lentiviral pHIV7-GFP vector. The following shRNA sequences were used: control shRNA, 5′-ACT CAA AAG GAA GTG ACA AGA-3′; METTL3 shRNA-1, 5′-GCT GCA CTT CAG ACG AAT T-3′ (Zhao et al., 2014); METTL3 shRNA-2, 5′-CCA CCT CAG TGG ATC TGT T-3′ (Dominissini et al., 2012); METTL14 shRNA-1, 5′-GCT AAA GGA TGA GTT AAT-3′; METTL14 shRNA-2, 5′-GGA CTT GGG ATG ATA TTA T- 3′ (Ping et al., 2014); ADAM19 shRNA-1, 5′-GGA AGA TTT AAA CTC CAT GAA G-3′; and ADAM19 shRNA-2, 5′-CAA AGT GTT CAA TGG ATG CAA C-3′. The METTL3-expressing lentiviral vector was prepared by subcloning the human METTL3 coding sequences from pcDNA3/FLAG-METTL3 (Liu et al., 2014) (Addgene plasmid 53739) into the CSC lentiviral vector (Shi et al., 2004). The METTL3 catalytic mutant (aa395-398, DPPW/APPA)- expressing lentiviral vector was prepared by sub-cloning the mutant human METTL3 sequences from pFLAG-CMV2-METTL3 (mutant) vector (Lin et al., 2016) into the CSC lentiviral vector.

### Viral Preparation and Transduction

Lentiviruses were prepared using 293T cells as described previously (Shi et al., 2004). To transduce GSCs, cells were dissociated for overnight culture and then incubated with lentivirus and 4 μg/mL polybrene (AmericanBio) for 24 hr.

### Immunostaining

GSC immunostaining was performed as previously described (Cui et al., 2016) using antibodies, including mouse anti-GFAP (1:1,000; Sigma; catalog no. G3893) and rabbit anti-Tuj1 (1:6,000; Covance; catalog no. PRB-435P).

### m^6^A Dot Blot Assay

GSCs were either maintained in sphere cultures or induced into differentiation for 1 week using 0.5% FBS (Sigma) together with 1 μM all-trans retinoic acid (Sigma). Total RNAs were isolated from these GSCs using Trizol reagent (Ambion). GSCs were transduced with lentivirus expressing METTL3 or METTL14 shRNA or METTL3 cDNA. 7 days after transduction, total RNA was extracted. mRNA was prepared from total RNA using the Dynabeads mRNA purification kit (Ambion, catalog no. 61006). The indicated amount of mRNAs was used for dot blot analysis using an antibody specific for m^6^A (1:1,000; Synaptic Systems; catalog no. 202003). The intensity of dot blot signal was quantified by ImageJ.

### RT-PCR

Total RNA isolated using Trizol reagent (Ambion) was subjected to reverse transcription performed using the Tetro cDNA synthesis Kit (BioLINE). RT-PCR reactions were performed using SYBR Green Master Mix (Thermo Scientific) on the Step One Plus Real-Time PCR instrument (Applied Biosystems). The following primers were used for RT-PCR: METLL3 forward (F), 5′-TCA GCA TCG GAA CCA GCA AAG-3′; METLL3 reverse (R), 5′-TCC TGA CTG ACC TTC TTG CTC-3′; METLL14 F, 5′-GTT GGA ACA TGG ATA GCC GC-3′; METLL14 R, 5′-CAA TGC TGT CGG CAC TTT CA-3′; CD44 F, 5′-TGA GCA TCG GAT TTG AGA CC-3′; CD44 R, 5′-TGT CAT ACT GGG AGG TGT TGG-3′; ADAM19 F, 5′-CCT GGA TGG ACA AGA GGA AG-3′; ADAM19 R, 5′-CTC AGC TTT GAG TGG ATG CT-3′; EPAH3 F, 5′-GGG CTG GAT CTC TTA TCC ATC-3′; EPAH3 R, 5′-GGA CCC AGT TTG TTC TCA GC-3′; KLF4 F, 5′-AAG AGT TCC CAT CTC AAG GC-3′; KLF4 R, 5′-CCG TGT GTT TAC GGT AGT GC-3′; and ACTIN F, 5′-CCG CAA AGA CCT GTA CGC CAA C-3′; and ACTIN R, 5′-CCA GGG CAG TGA TCT CCT TCT G-3′. ACTIN was included as the reference gene for normalization. The ΔΔCt method was used for quantification analysis.

### Cell Growth Assay

GSCs were transduced with lentivirus expressing METTL3 or METTL14 shRNA or METTL3 cDNA. 3 days later, the transduced cells were seeded at 5 × 10^4^ cells per well in 24-well plates and cultured for 7 days. Cell number was counted using a hemocytometer.

### Sphere-Formation Assay

The sphere-formation assay was performed as previously described (Cui et al., 2016). Briefly, 3 days after viral transduction, transduced cells were seeded at one cell per well in 96-well plates (for those associated with the limiting dilution assay) or 100 cells per well in 48-well plates and cultured for 2 weeks. The sphere number was counted under a microscope. The sphere-formation rate was defined as the percentage of sphere-forming cells out of the total number of starting cells.

### Limiting Dilution Assay

GSCs were transduced with relevant shRNA or METTL3-expressing lentivirus. The transduced GSCs were seeded at 1, 5, 10, 20, 50, and 100 cells per well into 96-well plates. The number of neurospheres in each well was counted 2 weeks after seeding cells. Extreme limiting dilution analysis was performed using software available at http://bioinf.wehi.edu.au/software/elda.

### Treatment of GSCs with the FTO Inhibitor MA2

GSCs were seeded at 5 × 10^4^ cells per well in 48-well plates and cultured overnight. These cells were treated with MA2, a chemical inhibitor of FTO (Huang et al., 2015), at 20, 40, 60, or 80 μM or with vehicle control and cultured for 48 hr. Cell number was counted using a hemocytometer. To determine the effect of MA2 on the level of m^6^A RNA modification, GSCs were treated with 50 μM MA2 for 48 hr. mRNAs were prepared and subjected to m^6^A dot blot assay. To test the effect of MA2 in the limiting dilution assay, GSCs were treated at 40 μM or with vehicle control for 2 weeks.

### Animals

All animal-related work was performed under Institutional Animal Care and Use Committee (IACUC) protocol 05050 approved by the City of Hope Institutional Animal Care and Use Committee. 6- to 8-week-old male and female NSG mice (from The Jackson Laboratory) were used in an age- and gender-matched manner. Sample size was determined based on a t test for two-group independent samples to reach power of 0.8 and a significance level of 0.05. p < 0.05 was considered statistically significant.

### Viral Transduction followed by Transplantation

PBT707, PBT003, or PBT726 cells expressing luciferase gene were transduced with lentivirus expressing control shRNA, METTL3 shRNA, or METTL14 shRNA. 1 week after virus transduction, cells were transplanted into the frontal lobes of brains of NSG mice by stereotaxic intracranial injection. Briefly, 2 × 10^5^ dissociated cells in 2 μL PBS were injected into the following site (anteroposterior [AP] +0.6 mm, mediolateral [ML] +1.6 mm, and dorsoventricular [DV] −2.6 mm) with a rate of 1 μL/min. Tumor growth was monitored by bioluminescence xenogen imaging every other week for 6–10 weeks. The bioluminescence intensity was quantified. When monitoring tumor growth, investigators were blinded to the group allocation during the bioluminescence xenogen imaging and aware of group allocation when assessing the outcome. The survival of mice after cell transplantation was recorded and analyzed.

### Intracranial Delivery of the FTO Inhibitor MA2

PBT003 cells (2 × 10^5^) transduced with luciferase expressing lentivirus were intracranially transplanted into the frontal lobe of NSG mice at the same coordinates as described above. 1 week after transplantation, tumors were detected by bioluminescence imaging and mice were treated with MA2 (5 μL of 600 μM MA2 in 1% DMSO in PBS per mouse) or vehicle control by intratumoral injection once a week for 4 weeks. Tumor growth was monitored by bioluminescence imaging every week for 6 weeks. The bioluminescence intensity was quantified.

### m^6^A-Seq

PBT003 cells were transduced with lentivirus expressing control shRNA or relevant shRNA. 7 days after transduction, total RNA was extracted using Trizol reagent (Ambion). mRNA was further purified using a Dynabeads mRNA purification kit (Ambion, catalog no. 61006). Fragmented RNA was subjected to m^6^A-immunoprecipitation (m^6^A IP) using anti-m^6^A rabbit polyclonal antibody (Synaptic Systems; catalog no. 202003) followed by RNA-seq. Further details of m^6^A-seq and data analysis are in Supplemental Experimental Procedures.

### Statistics

Statistical significance was analyzed using the unpaired one-tailed Student's t test. Values are presented as *p < 0.05, **p < 0.01, and ***p < 0.001. Error bars represent SD of the mean if not stated otherwise. A log-rank test was used for animal survival analysis.

## Supplementary Material

Sup.Info

## Highlights

- The self-renewal of GSCs is regulated by m^6^A RNA methylation
- Reduced m^6^A RNA methylation promotes the tumorigenesis of GSCs
- An FTO inhibitor suppresses the progression of GSC-initiated tumor
- m^6^A-seq reveals transcriptome-wide mRNA modification and regulation by m^6^A in GSCs
