# Supplementary material for: m6A RNA Methylation Regulates the Self-Renewal and Tumorigenesis of Glioblastoma Stem Cells
Source: Cell Rep. Author manuscript; Available in PMC 2018 Mar 14. (PMC5479356; doi:10.1016/j.celrep.2017.02.059)
Supplement: Sup.Info [file NIHMS866796-supplement-Sup_Info.pdf]

Cell Reports, Volume *18*

Supplemental Information

m^{6}A RNA Methylation Regulates the Self-Renewal and Tumorigenesis of Glioblastoma Stem Cells

Qi Cui, Hailing Shi, Peng Ye, Li Li, Qiuhao Qu, Guoqiang Sun, Guihua Sun, Zhike Lu, Yue Huang, Cai-Guang Yang, Arthur D. Riggs, Chuan He, and Yanhong Shi

SUPPLEMENTAL FIGURES AND LEGENDS

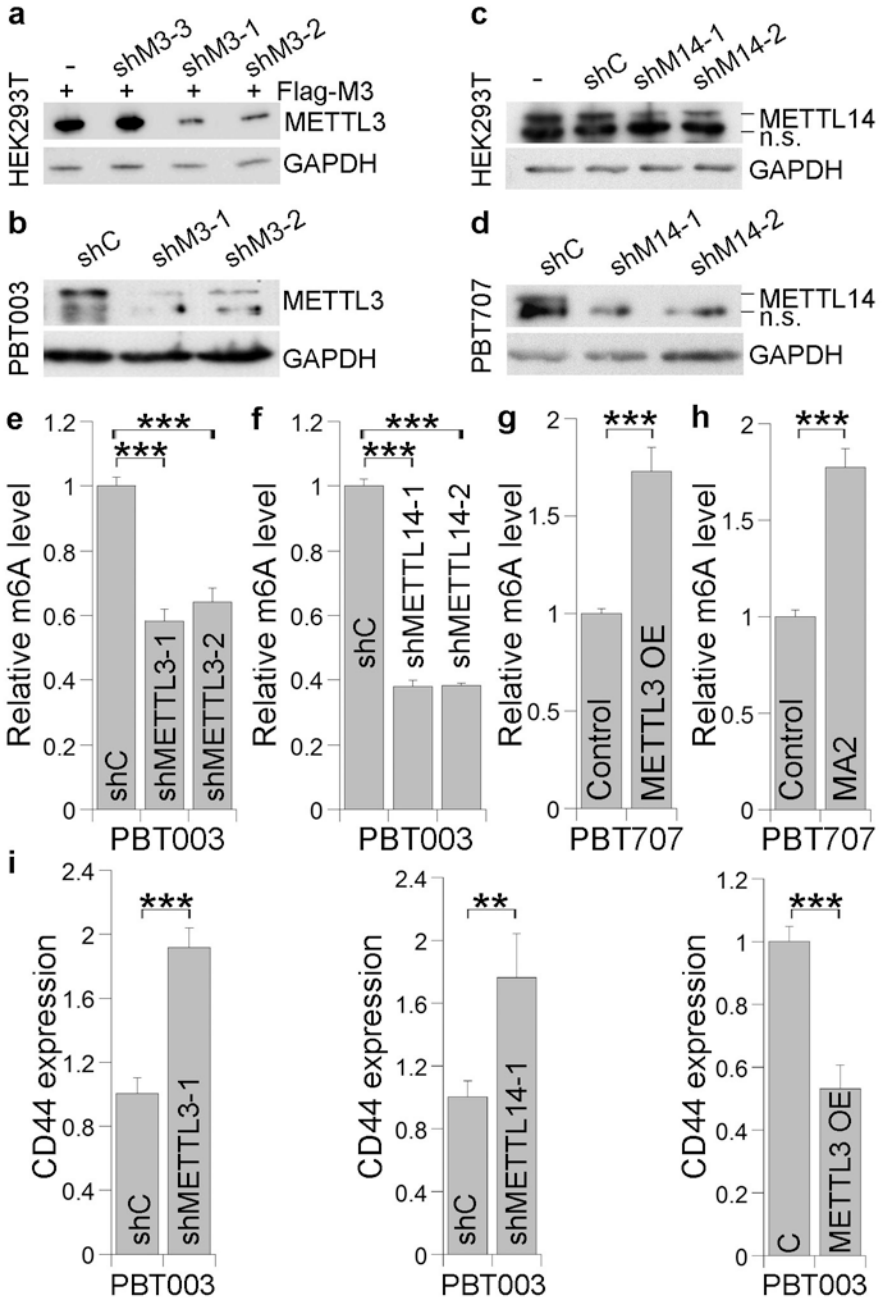

Figure S1 Modulation of m^{6}A mRNA modification by manipulating the m^{6}A methylation machinery in GSCs. Related to Figures 2, 3, 4, and 6. a, b. Western blot analysis of METTL3 KD in Flag-METTL3 (Flag-M3)-expressing HEK293T (**a**) and PBT003 (**b**) cells. **c, d.** Western blot analysis of METTL14 KD in HEK293T (**c**) and PBT707 (**d**) cells. **e, f.** mRNA dot blot analysis of m^{6}A levels in METTL3 or METTL14 KD PBT003 cells. shC: control shRNA; shMETTL3-1 and shMETTL3-2: shRNAs for METTL3; shMETTL14-1 and shMETTL14-2: shRNAs for METTL14. **g.** mRNA dot blot analysis of m^{6}A levels in PBT007 cells transduced with METTL3-expressing virus or control virus. **h.** mRNA dot blot analysis of m^{6}A levels in PBT007 cells treated with vehicle control or MA2. n=3. ***p<0.001 by Student's t-test. Error bars are s.e. of the mean. **i.** RT-PCR analysis of CD44 expression in PBT003 cells treated with control shRNA (shC), METTL3 shRNA 1 (shMETTL3-1), METTL14 shRNA 1 (shMETTL14-1) or METTL3 overexpression (OE). n=3. **p<0.01, ***p<0.001 by Student's t-test. Error bars are s.d. of the mean.

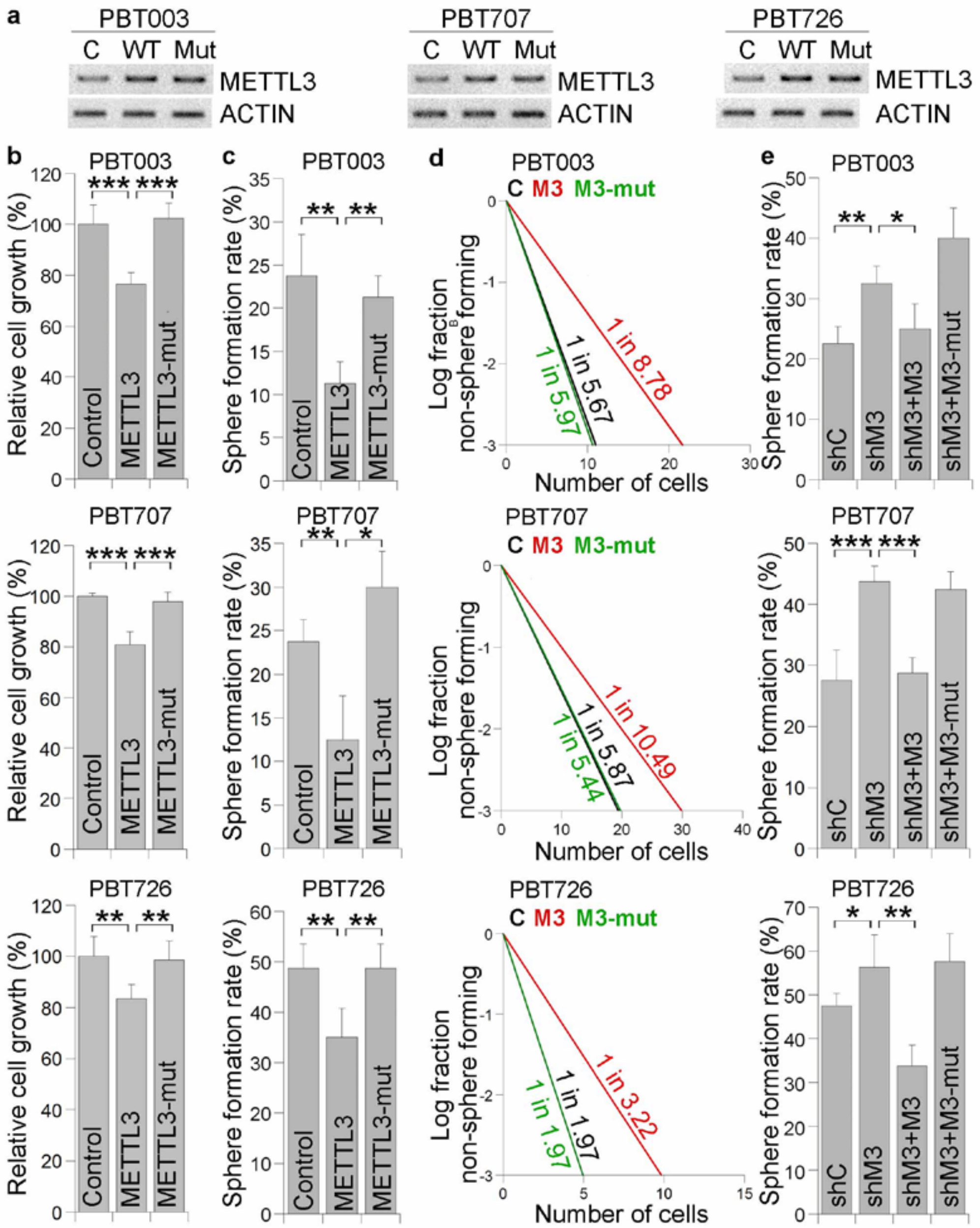

Figure S2 Overexpressing the wild type, but not catalytically inactive METTL3 inhibits the growth and self-renewal of GSCs. Related to Figure 4. a. RT-PCR analysis showing overexpression of the wild type (WT) or the catalytic mutant (Mut) METTL3 in GSCs (PBT003, PBT707, and PBT726 cells). **b-d.** Cell growth (**b**), sphere formation (**c**), and LDA (**d**) analyses of GSCs (PBT003, PBT707, and PBT726 cells) transduced with the control virus (C), the WT METTL3 (M3) or the catalytic mutant METTL3 (M3-mut)-expressing virus. **e.** Sphere formation assay of GSCs transduced with lentivirus expressing METTL3 shRNA (shM3) alone or together with the WT METTL3 (M3) or the catalytic mutant METTL3 (M3-mut). n=4 for **b**, **c, e**. n=20 for **d**. *p<0.05, **p<0.01, ***p<0.001 by Student's t-test. Error bars are s.d. of the mean.

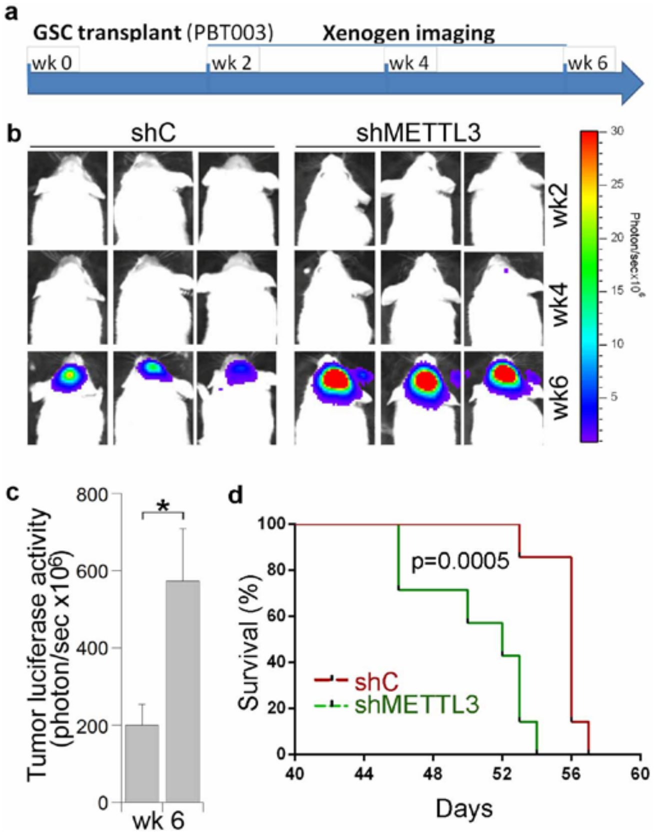

Figure S3 Knocking down METTL3 expression enhances the tumorigenicity of GSCs in PBT003 cells. Related to Figure 5. a. Schematic of the experimental design, including GSC transplantation and xenogen imaging of xenografted tumors. **b**. Xenogen images of brain tumors in NSG mice transplanted with PBT003 cells that were transduced with control shRNA (shC) or METTL3 shRNA (shMETTL3). The scale bar for bioluminescence intensity is shown on the right. **c**. Quantification of the bioluminescence intensity of tumors. *p<0.05 by Student's t-test. Error bars are s.d. of the mean. **d.** The survival curves of NSG mice transplanted with PBT003 cells transduced with control shRNA (shC) or METTL3 shRNA (shMETTL3). The X axis represents days after GSC transplantation. n=7, log-rank test.

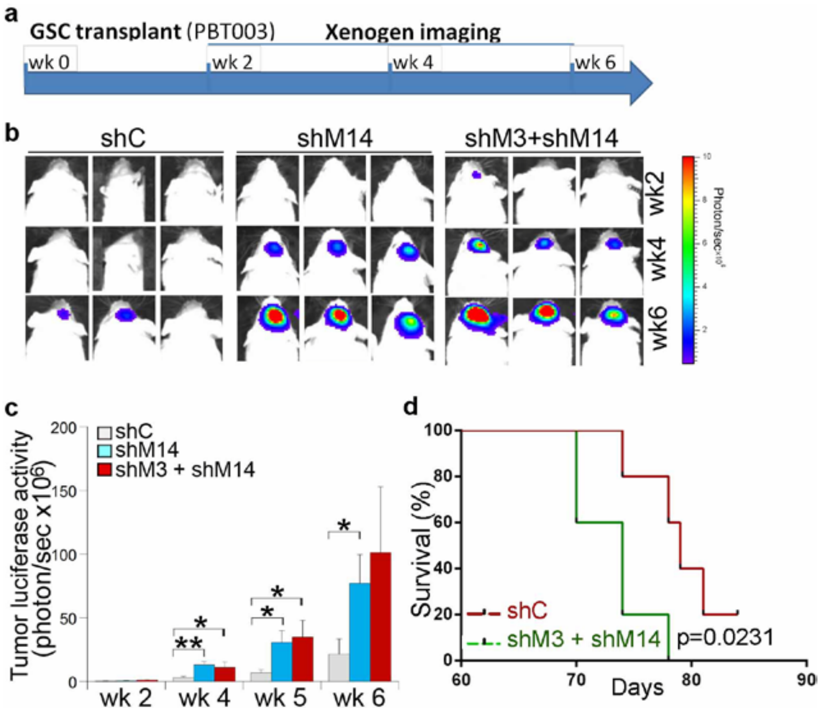

Figure S4 Knocking down METTL3 and METTL14 expression promotes the tumorigenicity of GSCs in PBT003 cells. Related to Figure 5. a. Schematic of the experimental design, including GSC transplantation and xenogen imaging of xenografted tumors. **b**. Xenogen images of brain tumors in NSG mice transplanted with PBT003 cells that were transduced with control shRNA (shC), METTL14 shRNA (shM14), or the combination of METTL14 shRNA and METTL3 shRNA (shM3 + shM14). The scale bar for bioluminescence intensity is shown on the right. **c.** Quantification of the bioluminescence intensity of tumors in NSG mice transplanted with PBT003 cells that were transduced with shC, shM14, or shM3 + shM14. *p<0.05, **p<0.01 by Student's t-test. Error bars are s.d. of the mean. **d**. The survival curves of NSG mice transplanted with PBT003 cells transduced with shC or shM3 + shM14. The X axis represents days after GSC transplantation. n=5, log-rank test.

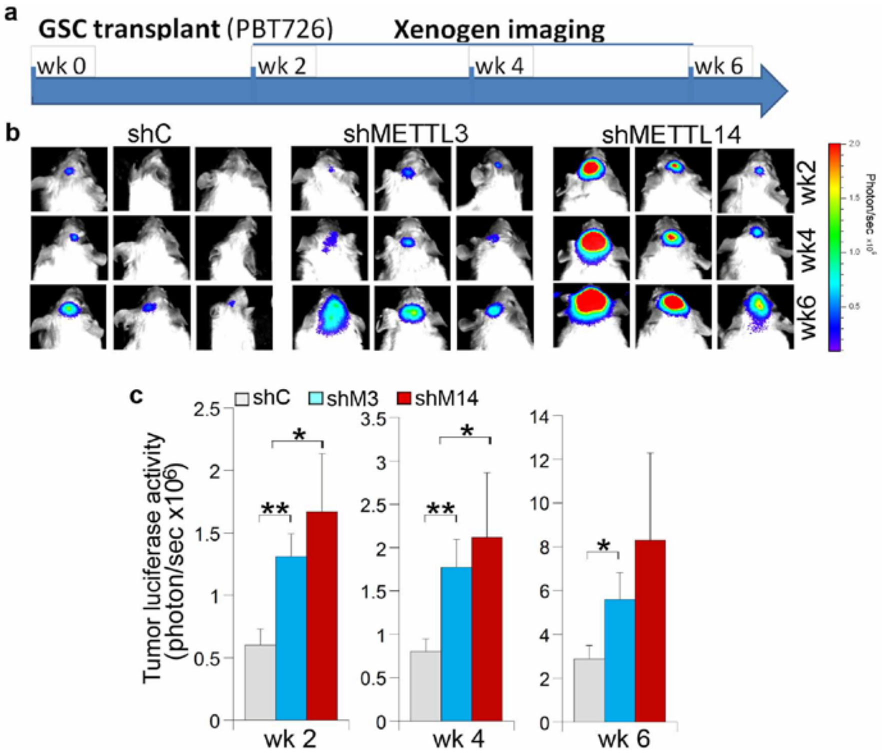

Figure S5 Knocking down METTL3 or METTL14 expression increases the tumorigenicity of GSCs in PBT726 cells. Related to Figure 5. a. Schematic of the experimental design, including GSC transplantation and xenogen imaging of xenografted tumors. **b**. Xenogen images of brain tumors in NSG mice transplanted with PBT726 cells that were transduced with control shRNA (shC), METTL3 shRNA (shMETTL3) or METTL14 shRNA (shMETTL14). The scale bar for bioluminescence intensity is shown on the right. **c.** Quantification of the bioluminescence intensity of tumors in NSG mice transplanted with PBT726 cells that were transduced with control shRNA (shC), METTL3 shRNA (shM3) or METTL14 shRNA (shM14). n=8, *p<0.05, **p<0.01 by Student's t-test. Error bars are s.d. of the mean.

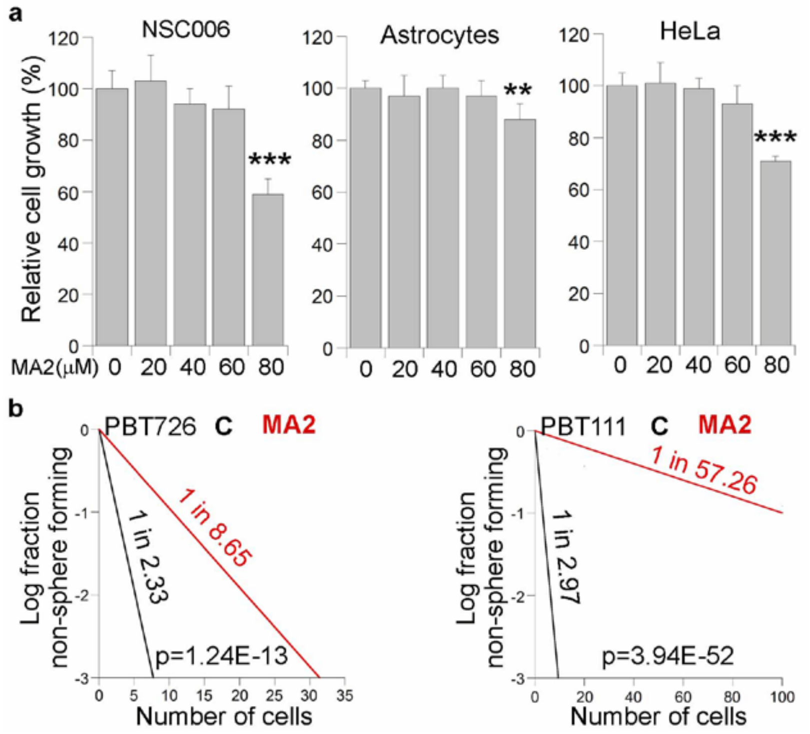

Figure S6 The FTO inhibitor MA2 suppresses the growth and self-renewal of GSCs. Related to Figure 6. a. Cell growth analyses of neural stem cells (NSC 006), astrocytes, and HeLa cells treated with the FTO inhibitor MA2. NSC006, astrocytes and HeLa cells were included as controls for GSCs shown in Fig. 6a. n=4. **b.** LDA analysis of GSCs treated with MA2 or vehicle control. n=20. ***$p<0.001$ by Student's t-test. Error bars are s.d. of the mean.

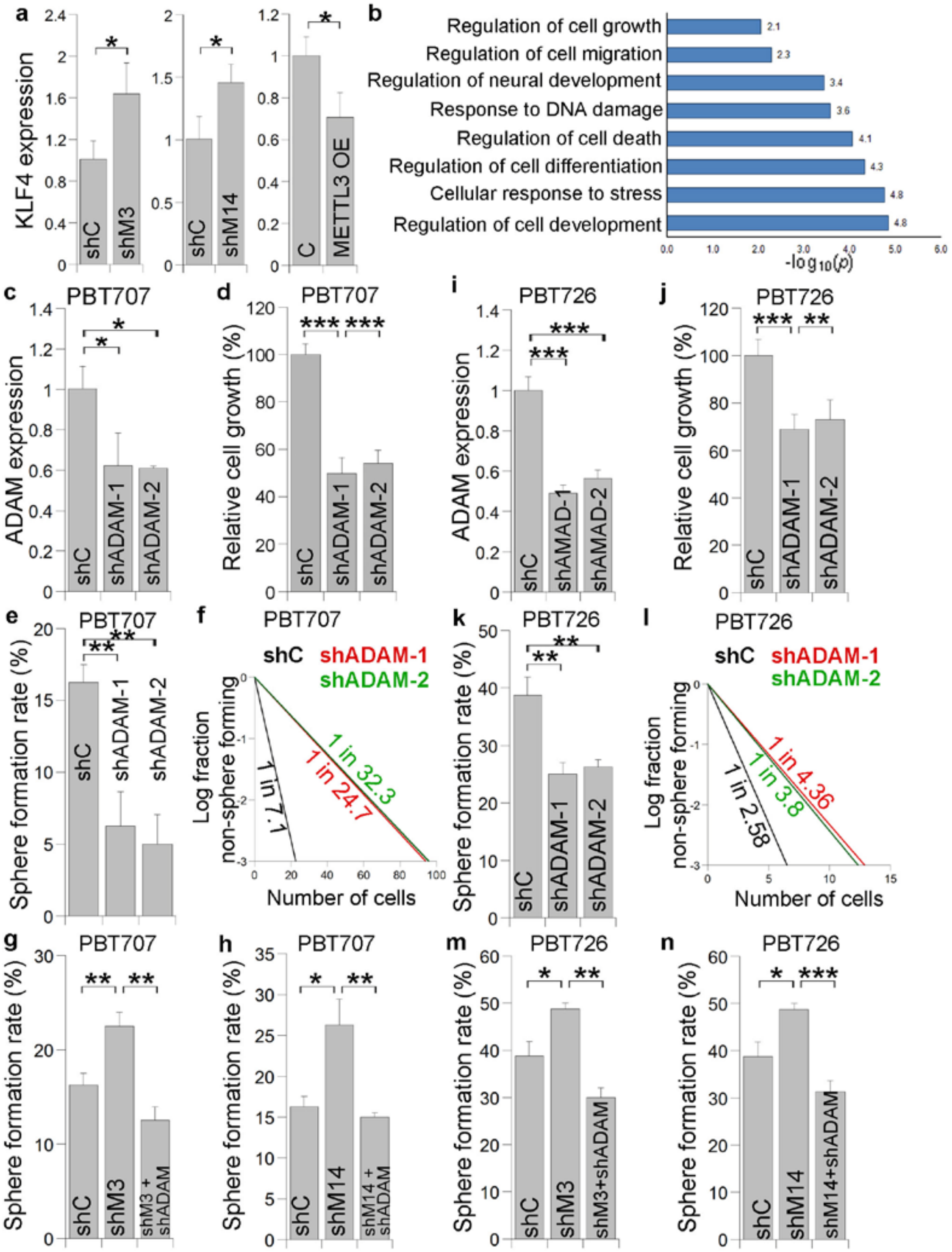

Figure S7 METTL3 or METTL14 KD induces mRNA expression and m[6]A methylation level changes in GSCs. Related to Figure 7. a. RT-PCR analysis of KLF4 expression in PBT003 cells treated with control shRNA (shC), METTL3 shRNA (shM3), METTL14 shRNA (shM14), or METTL3 overexpression (OE). n=3. *p<0.05 by Student's t-test. Error bars are s.d. of the mean. **b.** GO analysis of transcripts with m[6]A peaks in GSCs. **c, i.** RT-PCR analysis of ADAM19 (ADAM) expression in GSCs transduced with lentivirus expressing control shRNA (shC) or ADAM19 shRNAs (shADAM-1, shADAM-2). n=3. **d-f, j-l.** Cell growth (**d, j**), sphere formation (**e, k**), and LDA (**f, l**) analyses of GSCs (PBT707 and PBT726 cells) transduced with lentivirus expressing control shRNA (shC) or ADAM19 shRNAs (shADAM-1, shADAM-2). n=4 for **d**, **e**, **j**, **k**. n=20 for **f, l**. **g-h & m-n.** Sphere formation analyses of PBT707 (**g, h**) and PBT726 (**m, n**) cells transduced with lentivirus expressing METTL3 shRNA (shM3) or METTL14 shRNA (shM14) alone or together with ADAM19 shRNA (shADAM). n=4. *p<0.05, **p<0.01, ***p<0.001 by Student's t-test. Error bars are s.d. of the mean.

Supplemental Experimental Procedures

Western blot analysis

Twenty µg proteins from the whole cell lysates were used for Western blot analysis. Rabbit anti-METTL3 antibody (1:1000; Proteintech; Catalog # 15073-1-AP), rabbit anti-METTL14 antibody (1:500; Sigma; Catalog# HPA038002), and rabbit anti-GAPDH antibody (1:1000; Santa Cruz Biotechnology; Catalog # sc-25778) were used.

m^6A-seq

Purified mRNA samples from PBT003 cells were used for m^6A-seq. RNA fragmentation was performed by sonication at 10 ng μl^{-1} in 100 µl RNase-free water using Bioruptor Pico (Diagenode) with 30 s on / 30 s off cycle for 30 cycles. m^6A-immunoprecipitation (m^6A IP) and library preparation were performed according to a published protocol (Dominissini et al., 2013). In detail, 2.5 µg affinity purified anti-m^6A rabbit polyclonal antibody (Synaptic Systems; Catalog # 202003) and 20 µl Protein A beads (ThermoFisher; Catalog# 10002D) were used for each affinity pull-down. m^6A antibody-bound RNAs were eluted with 100 µl elution buffer and recovered by RNA Clean and Concentrator-5 (Zymo), and subjected to RNA library preparation with TruSeq Stranded mRNA Library Prep Kit. Sequencing was carried out on Illumina HiSeq 4000 according to the manufacturer's instructions.

m^6A-seq data analysis

m^6A seq samples (inputs and m^6A-IPs) were sequenced by Illumina HiSeq 4000 with single end 50-bp read length. The adapters were trimmed by using the FASTX-Toolkit (Pearson et al., 1997). Deep sequencing data were mapped to Human genome version hg38 using Tophat version 2.0 (Trapnell et al., 2009) without any gaps and allowed for at most two mismatches. Input samples were analyzed by Cufflink (v2.2.1) (Trapnell et al., 2010) to generate RPKM (reads per kilobase, per million reads). For input and m^6A-IP samples, the longest isoform was used if the gene had multiple isoforms. Aligned reads were extended to 150 bp (average fragments size) and converted from genome-based coordinates to isoform-based coordinates, in order to eliminate the interference from introns in peak calling. The peak calling method was modified from published work (Dominissini et al., 2012). To call m^6A peaks, the longest isoform of each gene was scanned using a 100 bp sliding window with 10 bp step. To reduce bias from potential inaccurate gene structure annotation and the arbitrary usage of the longest isoform, windows with read counts less than 1/20 of the top window in both m^6A-IP and input sample were excluded. For each gene, the read counts in each window were normalized by the median count of all windows of that gene. A Fisher exact test was used to identify the differential windows between m^6A-IP and input samples. The window was called as positive if the FDR < 0.01 and log2 (Enrichment Score) ≥ 1. Overlapping positive windows were merged. The following four numbers were calculated to obtain the enrichment score of each peak (or window): reads count of the m^6A-IP samples in the current peak/window (a), median read counts of the m^6A-IP sample in all 100 bp windows on the current mRNA (b), reads count of the input sample in the current peak/window (c), and median read counts of the input sample in all 100 bp windows on the current mRNA (d). The enrichment score of each window was calculated as $(a \times d)/(b \times c)$. The enrichment ratio was calculated as the ratio of enrichment score in two samples. For motif analysis, HOMER (Heinz et al., 2010) was used to search motifs in each set of m^6A peaks. The longest isoform of all genes was used as background. For m^6A peak distribution analysis, the length of the 5′ UTR, CDS, and 3′ UTR of each gene was normalized into 50 bins, and the normalized peak density in each bin was calculated as the percentage of gene that has m^6A peak in that bin. The gene ontology analysis was performed using the DAVID database with biological process classified under default settings.

Supplemental References

Heinz, S., Benner, C., Spann, N., Bertolino, E., Lin, Y.C., Laslo, P., Cheng, J.X., Murre, C., Singh, H., and Glass, C.K. (2010). Simple combinations of lineage-determining transcription factors prime cis-regulatory elements required for macrophage and B cell identities. Molecular cell *38*, 576-589.

Pearson, W.R., Wood, T., Zhang, Z., and Miller, W. (1997). Comparison of DNA sequences with protein sequences. Genomics *46*, 24-36.

Trapnell, C., Pachter, L., and Salzberg, S.L. (2009). TopHat: discovering splice junctions with RNA-Seq. Bioinformatics *25*, 1105-1111.

Trapnell, C., Williams, B.A., Pertea, G., Mortazavi, A., Kwan, G., van Baren, M.J., Salzberg, S.L., Wold, B.J., and Pachter, L. (2010). Transcript assembly and quantification by RNA-Seq reveals unannotated transcripts and isoform switching during cell differentiation. Nature biotechnology *28*, 511-515.
